# Supplementary material for: Effect of scanning-aid agents on the scanning accuracy in specially designed metallic models: A laboratory study
Source: PLoS One. 2022 May 5;17(5):e0267742. doi: 10.1371/journal.pone.0267742 (PMC9070957; doi:10.1371/journal.pone.0267742)
Supplement: Attachment — Submitted filename: Y-J Lim Response to Reviewers.docx [file pone.0267742.s001.docx]

Dear editor

Manuscript Number: PONE-D-21-40935

“Effect of Scanning-Aid Agents on the Scanning Accuracy in Specially Designed Metallic Models: A Laboratory Study"

We thank the reviewers for their comments, and we are grateful for the opportunity to provide further revisions to our paper. We changed our manuscript according to the reviewers’ comments and recommendations. We are trying to adequately address each of the points made by the reviewers. We would be very thankful if you could please reconsider a thoroughly revised manuscript. We highlighted the changes made in the manuscript by using a different color font (red): see correction marked form file, and explained details in this letter.

**Response to Reviewer #1 Comments**

The authors submit an article "Effect of Scanning-Aid Agents on the Scanning Accuracy in Specially Designed Metallic Models: A Laboratory Study". The study compares three types of sprays that are applied to the metal surface of dental prostheses and subsequently scanned with an intraoral scanner. Is there often a need in dental practice to scan metal surfaces of prostheses with an intraoral scanner? What other significant difference is there used between sprays (other than liquid type and powder type)?

**Response:** Thank you for your valuable comment. Scanning-aid materials used in this study are often necessary in daily clinical practice because the metallic shiny surface of prostheses(ex; metal crown or restoration) prevent the intraoral scanner from recognizing and obtaining the surface images properly. Composition and color are different between scanning-aid materials. According to each product information, ScanCure and IP Scan Spray contain titanium dioxide as their main components and have a white color, whereas VITA Powder Scan Spray is a spray-on, titanium dioxide free and blue colored pigment suspension.

The work scans the metal surface of prostheses - wouldn't it be enough to sandblast the surface to eliminate the shiny surface?

**Response:** Thank you for your valuable comment. The dental sandblasting is usually used to increase bonding strength during the cementation of prosthesis by mechanically roughening the surface. However, if it is used to eliminate the shiny surface of prosthesis in patient’s mouth, it adversely affects the polished surface of prosthesis which is essential for oral hygiene care. Also, the powder in sandblasting rather than the specialized scanning-aid agents can cause more discomforts for patients.

3. Cobalt chromium alloy or titanium alloy, is usually used in dental practice. Why did the authors use an aluminum alloy? It has no surface properties other than cobalt chromium alloy or titanium alloy?

**Response:** Thank you for your valuable comment. Due to the limitation of material selection in making the specially designed metallic models, we are focused on simulating the shiny surface of metallic model which interferes the scanner’s recognition of scanned images

As you mentioned, further studies with cobalt chromium alloy or titanium alloy in real patient’s mouth are necessary to reflect the real clinical situation.

4. In the article, you state that you used the I500 intraoral scanner for scanning - what technology does it scan with? Optical light? Laser? What is the accuracy and speed of scanning? Have you taken into account Trueness and Precision of the intraoral scanner itself when evaluating the data obtained?

**Response:** Thank you for your valuable comment. The I500 intraoral scanner uses video-type scanning based on triangulation technology with optical light. The accuracy is described by two measurement methods: trueness and precision. Trueness refers to the deviation of the measured value from the original value whereas precision refers to the closeness between repeated measured value. The speed of scanning was evaluated by measuring the time to obtain the total scanned image. We have considered the trueness and precision of the intraoral scanner by the product information which is based on the experiments from their own company and several published journals. Further studies using other types of intraoral scanners such as confocal-type type (e.g., 3Shape Trios®) are necessary to verify the accuracy between intraoral scanners.

5. The article is clearly written, described in detail Materials and Methods. The Trueness and Precision data are evaluated in the Results chapter. In Conclusion, it is stated that it is better to use a liquid type spray. This is certainly important information for dental practice, but I still miss the point, the main idea of the article. Is it a comparison of sprays or a comparison of metallic reference models (inlay, onlay, and bridge)? Why is it important for the dental technician / dentist which spray is better?

**Response:** Thank you for your valuable comment. The main point of this article is that how different types of scanning-aid materials affect the scanning accuracy using an intraoral scanner in specially designed metallic models. Inlay, onlay and bridge models were individually evaluated because we wanted to know if the same results of accuracy of scanning-aid materials were obtained in the cases of different shapes and distances. Particularly, in the bridge model, there were significantly differences of the trueness among the three different scanning-aid materials. It is important for the dental staff to know which spray is better because the accuracy of scanned image affects the success of the long-term prognosis of prosthesis

**Response to Reviewer #2 Comments**

The report is well written and easy to understand. Accurate results have been achieved. The information is also accurately illustrated. The authors work with modern technologies in design. Although the SolidWorks software is not the latest version (version 2016 is listed), it is strong enough functionally.

**Response:** Thank you for your favorable review for our manuscript.

Not as a remark, but as a recommendation:

- If possible, use institutional contact emails.

**Response:** Thank you for your valuable comment. “limdds@snu.ac.kr and bjkim016@snu.ac.kr” is the email of the Seoul National University.

- The conclusion is too small. It should increase the size. (Also in conclusion) The most important points should be noted in separate paragraphs.

**Response:** Thank you for your valuable comment. The following sentence was separated from the original paragraph as suggested by the reviewer.

Line 248: “Based on our findings, we recommend that liquid-type scanning agents should be used to obtain more accurate scan images of the metallic surfaces of dental restorations in clinical practice.”

**Response to Reviewer #3 Comments**

This is a fascinating study about the effect of scanning-aid agents on scanning accuracy in specially designed metallic models. Hence, the study adds to the current knowledge in this relatively new field and is definitely of clinical significance. However, it is still not clear to me the following matters as far as I read the manuscript.
This study aimed to evaluate the effect of different scanning-aid agents on the scanning performance of intraoral scanners. However, the description of ScanCure in the test group is insufficient. It is a liquid, so the authors need to describe more about it, including the ingredients. Also, there should be an explanation of how the agent from each group is applied. In what order and how many times these were applied or sprayed on the model. It is also necessary to describe how much powder was sprayed each time. The distance and angulation of the spray tip from the specimen are essential.

**Response:** Thank you for your valuable comment. The following paragraphs were added in materials and methods section as suggested by the reviewer.

Line 106: “VITA Powder Scan Spray is a spray-on, titanium dioxide free and blue colored pigment suspension with ethanol and isobutane. IP Scan Spray contains titanium dioxide with ethanol, propane, butane, and isobutane. And, ScanCure contains titanium dioxide and ethanol.”

Line 110: “Because the application procedure of scanning-aid materials is sensitive to operator’s proficiency, one skilled prosthodontist applied the materials on the model as follows: the liquid type of ScanCure was applied by one brush stroke at a time in all surfaces of model, powder type of VITA and IP scan spray was applied at a same distance (5 inch) and angulation (45 degree) of the spray tip from the specimen with the same time (2 seconds) to make an uniform thin layer of powder.”

In the 'Acquisition of Digital Data' section, the authors say that it was difficult to obtain a reference dataset, so it was obtained using 3D design, but it is not easy to understand. First of all, HERO7106 equipment is a contact scanner known as a Coordinate Measuring Machine (CMM), not a laser scanner. As I understand it, to scan with a laser scanner, the spray must be applied no matter how industrial-level accurate scanners are, so the quality of scan data is no different from that of the test group intraoral scanner. By reflecting the dimensions of each block measured by CMM in the design data (mentioned as 3D design in the manuscript), the data was transformed into the same size as the real milled. And then, this modified data was used as reference data. I want to check whether I understood well. In addition, no matter how accurate measured values are reflected as design data, it is thought that there might still be errors. How did the authors try to overcome this part?

**Response:** Thank you for your valuable comment. It is clearly correct what you understood as mentioned above. We wanted to make the reference dataset which was not affected by the scanning-aid materials. Of course, there should be inevitable error because it was not obtained by direct scanning. However, we try to reduce the error as possible by reflecting many measured data in the designing software.

Tables 1 and 2 do not contain information on statistical results. In the discussion section, the authors mentioned trueness, but more analysis on precision results should follow. Although there was no statistical difference, the ScanCure group showed high precision, which should be added to the manuscript. The first sentence of the second paragraph in the discussion section says '~ VITA group had lowest RMS values,' but it seems to be the opposite. In the middle of the third paragraph, 'A hardware design of the products was sufficient to reproduce the results,' it is unclear whether the products refer to sprays/liquid, intraoral scanner, or specimen, so it is difficult to understand the intention of the authors.

**Response:** Thank you for your valuable comment. Fig 4 and 5 are box plots which include data in Table 1 and 2 respectively.

As confirmed in Fig 5 and Table 2, the IP group showed the highest precision among the three groups with all the reference models although there was no statistical difference. The following sentence was revised in the discussion section.

Line 221: “Unlike trueness, there were no statistically significant differences in the RMS values of precision even though the IP group showed the highest precision among the three groups with all the reference models.”

The following sentence was revised to correct our mistake.

Line 204: “Furthermore, the VITA group had the largest RMS values among the three groups with all the reference models, with a statistically significant difference.”

The products what we meant were scanning-aid materials. The following sentence was revised to clarify our intention.

Line 225: “However, in each group, the hardware design of the scanning-aid materials was sufficient to reproduce the results of the scanned images.”
